# Demineralized dentin matrix promotes gingival healing in alveolar ridge preservation of premolars extracted for orthodontic reason: a split-mouth study

**DOI:** 10.3389/fendo.2023.1281649

**Published:** 2023-10-19

**Authors:** Xiaofeng Xu, Dongsheng Peng, Bowei Zhou, Kaijin Lin, Siyi Wang, Wei Zhao, Minqian Zheng, Jin Yang, Jianbin Guo

**Affiliations:** ^1^Fujian Provincial Engineering Research Center of Oral Biomaterial, Fujian Medical University, Fuzhou, China; ^2^School and Hospital of Stomatology, Fujian Medical University, Fuzhou, China; ^3^Department of Stomatology, Affiliated Hospital of Putian University, Putian, China; ^4^Department of Stomatology, Fujian Maternity and Child Health Hospital, Affiliated Hospital of Fujian Medical University, Fuzhou, China; ^5^Department of Stomatology, Fujian Obstetrics and Gynecology Hospital, Fuzhou, China; ^6^Research Center of Dental and Craniofacial Implants, Fujian Medical University, Fuzhou, China

**Keywords:** demineralized dentin matrix, alveolar ridge preservation, gingival healing, bone graft materials, orthodontics

## Abstract

**Objective:**

The purpose of this study was to prospectively evaluate the efficacy of a demineralized dentin matrix (DDM) in decreasing the initial inflammatory response of the gingiva and facilitating the repair and regeneration of soft tissue in alveolar ridge preservation.

**Methods:**

This clinical study employed a split-mouth design. Fourteen patients with a total of forty-four sites underwent extraction and alveolar ridge preservation (ARP) procedures. A Bilaterally symmetrical extraction operation were conducted on the premolars of each patient. The experimental group received DDM as a graft material for ARP, while the control group underwent natural healing. Within the first month postoperatively, the pain condition, color, and swelling status of the extraction sites were initially assessed at different time points Subsequently, measurements were taken for buccal gingival margin height, buccal-lingual width, extraction socket contour, and the extraction socket area and healing rate were digitally measured. Additionally, Alcian Blue staining was used for histological evaluation of the content during alveolar socket healing.

**Results:**

Both groups experienced uneventful healing, with no adverse reactions observed at any of the extraction sites. The differences in VAS pain scores between the two groups postoperatively were not statistically significant. In the early stage of gingival tissue healing (3 days postoperatively), there were statistically significant differences in gingival condition and buccal gingival margin height between the two groups. In the later stage of gingival tissue healing (7, 14, and 30 days postoperatively), there were statistically significant differences in buccal-lingual width, extraction socket healing area, and healing rate between the two groups. Furthermore, the histological results from Alcian Blue staining suggested that the experimental group may play a significant role in promoting gingival tissue healing, possibly by regulating inflammatory responses when compared to the control group.

**Conclusion:**

The application of DDM in alveolar ridge preservation has been found to diminish initial gingival inflammation after tooth extraction. Additionally, it has shown the ability to accelerate early gingival soft tissue healing and preserve its anatomical contour.

**Clinical trial registration:**

chictr.org.cn, identifier ChiCTR2100050650.

## Introduction

After tooth extraction, the loss of both soft and hard tissue contours at the extraction site can occur due to normal physiological remodeling and the absence of stimuli from chewing function (1, 2), This can have negative impacts on subsequent treatments such as implant therapy (3), orthodontic treatment (4, 5), and removable denture restoration (6, 7). Alveolar ridge preservation (ARP) refers to a series of treatment methods wherein, immediately after tooth extraction, bone grafting or the use of biological materials is performed within the extraction socket. This approach aims to slow down alveolar bone resorption, thereby effectively maintaining the existing soft and hard tissue contours to the maximum extent possible (8).

In previous studies of ARP, a wide range of biological materials were employed, including autogenous bone, allografts, xenografts, autologous blood-derived products, and bioactive agents (9, 10). Although autogenous bone possesses osteogenic, osteoinductive, and osteoconductive characteristics and is considered the “gold standard” of bone grafting materials (11), obtaining autogenous bone requires creating a secondary surgical site, which may lead to complications at the donor site (12). Allogeneic bone grafts can result in immune rejection reactions and the spread of diseases (13); furthermore, xenogeneic bone grafts have disadvantages such as high costs and a lack of capacity to promote early ossification and bone induction (14). Therefore, the search for cost-effective bone graft materials with superior material properties remains a focal point of concern in the current field of oral medicine.

Currently, demineralized dentin matrix (DDM) derived from extracted teeth is an emerging bone graft material with high biocompatibility (15). Since its first systematic report in 2008, the effectiveness and safety of DDM in bone augmentation procedures have been demonstrated through numerous animal experiments and clinical studies (16), Preliminary clinical research conducted by our research group has also confirmed the efficacy of DDM in bone regeneration (15). The inorganic components in DDM can serve as a scaffold to maintain space and volume, promoting donor cell attachment and proliferation, thus conferring osteoconductive properties (17). The organic components, on the other hand, supply a variety of growth factors that facilitate bone reconstruction and repair, creating an ideal environment for cellular differentiation and proliferation, thereby imparting osteoinductive properties (18).

During the initial stage of tooth extraction wound healing, the migration of fibroblasts is a fundamental component of wound contraction, and the expression of myofibroblast-related genes plays a crucial role in the early stages of healing (19). It is worth noting that recent studies by Bernardi (20) and Bianchi (21) have further revealed a positive response of DDM in inducing proliferation, adhesion, and migration of human periodontal ligament fibroblasts, indicating that DDM has the potential to promote the growth of human periodontal ligament fibroblasts cells. Therefore, DDM is not only effective in bone augmentation but also may have a potential promoting effect on soft tissue healing. However, in previous studies utilizing DDM for ARP, most of the research has focused on evaluating its effectiveness in bone augmentation, with a lack of comprehensive systematic reports on soft tissue healing aspects.

Based on previous research, this study hypothesizes that the application of DDM in ARP can promote early healing of the gingival tissue at the extracted tooth site. This split-mouth study involves the extraction of premolars required for orthodontic treatment. Immediately adjacent to the chairside, DDM is prepared and promptly filled into the extraction socket. The aim is to observe the potential promoting effects of DDM in ARP on gingival healing at the extracted tooth site.

## Methods

### Study design and bioethical considerations

This was a single-center, parallel-group, split-mouth design trial with balanced randomization (1:1) conducted at the Department of Oral and Maxillofacial Surgery, Affiliated Stomatological Hospital of Fujian Medical University from September 2020 to March 2022.

Recruiting patients from orthodontics requiring extraction of premolars to participate in this study. The primary researcher screened and recruited the patients. In the clinical research process, uninvolved researchers were tasked with using a coin-tossing method to select the experimental and control groups, where “heads” represented the experimental group and “tails” represented the control group.

The study followed the principles of the Declaration of Helsinki for research involving human subjects, and all participants provided written informed consent. Approval was obtained from the institutional review board of the School and Hospital of Stomatology, Fujian Medical University (Fujian, China). The trial was registered in the Chinese Clinical Trial Registry on 01/09/2021, which is a member of the World Health Organization’s international clinical trials registry, under the Registry Number ChiCTR2100050650. No significant changes were made to the trial design after the study had commenced.

### Inclusion and exclusion criteria

Inclusion criteria (1): Orthodontic patients (over 12 years old) who need symmetrical extraction of homonymous maxillary premolars (2); The patients had symmetrical left and right occlusion and alveolar bone (3); There is no bone metabolism disease such as diabetes and osteoporosis (4); Healthy periodontal tissue, no bone destruction such as cysts and tumors, and no history of orthodontic treatment (5); Preoperative blood tests indicated normal coagulation function and platelet count (6); The patient had no smoking history, no pregnancy, and good compliance.

Exclusion criteria (1): Those suffering from any systemic diseases (2); History of trauma or surgery, fracture or even loss of alveolar bone (3); Systemic diseases or drug application that may affect the healing of soft and hard tissues.

### Sample size calculation

The sample size was determined for each of the two groups, with 22 tooth extraction sites in each group. It was determined by ensuring a test power of at least 85% and a significance level of no more than 5% (using G*Power 3.1.9.2 software from Dusseldorf University). This calculation of sample size was based on the effect size estimation derived from a previously published research study (22).

### Procedures

**Figure 1:**
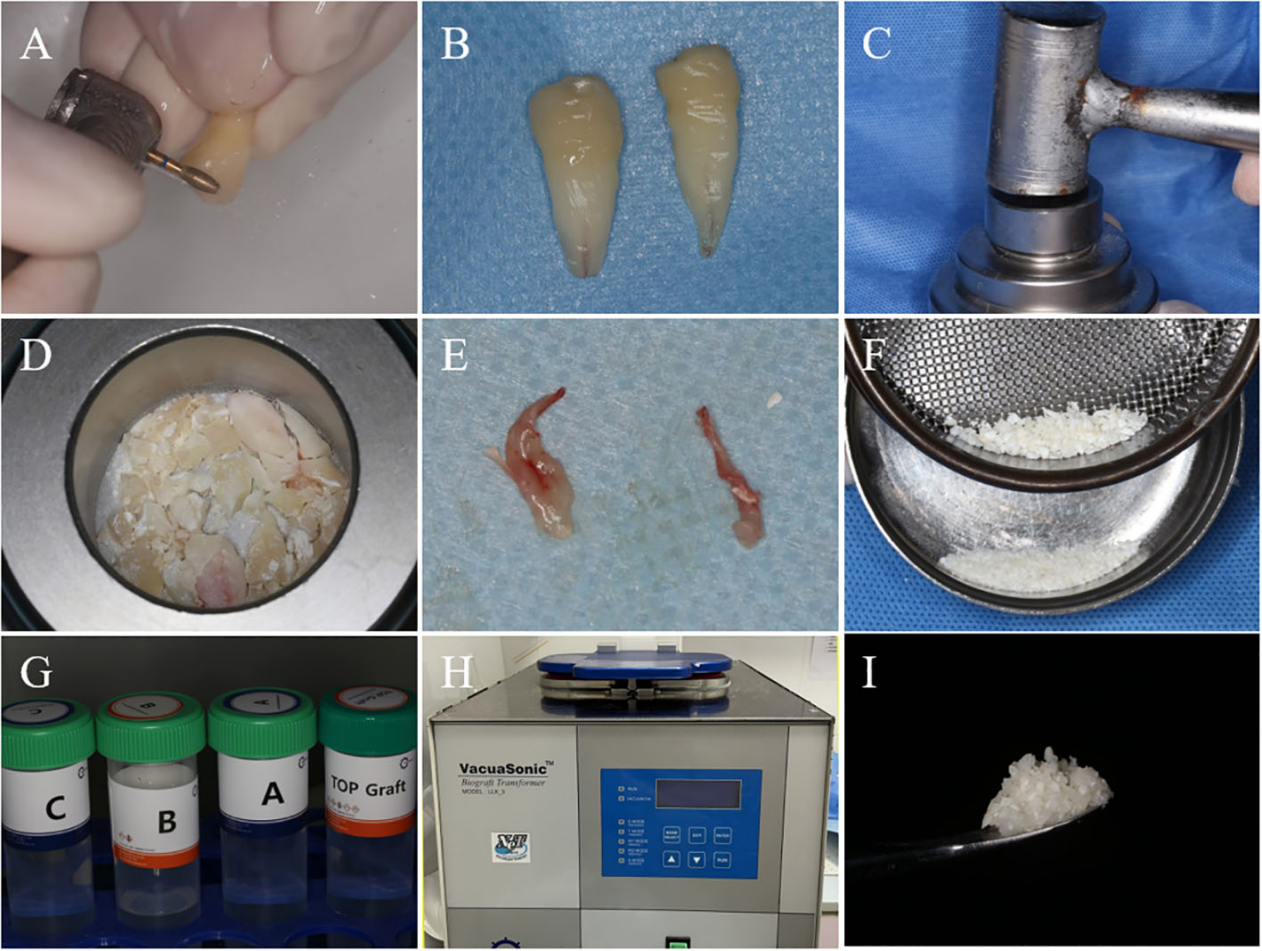
The process of DDM preparation. **(A)** After tooth extraction, the enamel and cementum of the teeth are removed using a high-speed handpiece. **(B)** Teeth after the removal of enamel and cementum. **(C)** Powder Kit tool for grinding dental tissues. **(D)** Dental tissue after initial grinding. **(E)** Removed dental pulp tissue. **(F)** After grinding dental tissue, filter it through a 1mm sieve to obtain dentin particles with a diameter smaller than 1mm. **(G)** The DecalSi^®^ PDM reagent used for demineralization, washing, and sterilization of dentin particles. **(H)** Using the VacuaSonic^®^ System device for programmed treatment of dentin particles. **(I)** DDM obtained after processing.

**Figure 2:**
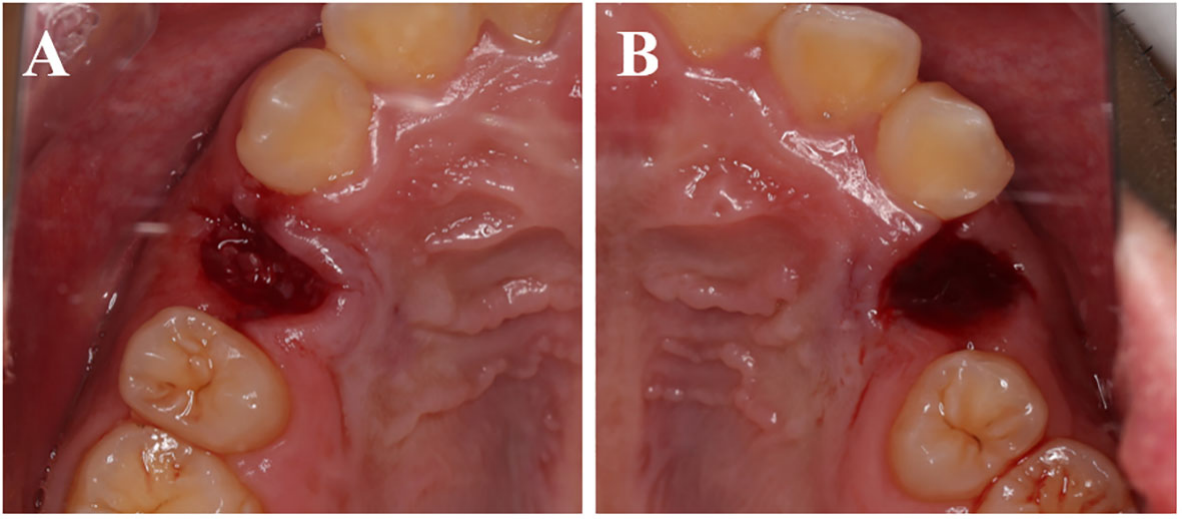
Immediate postoperative. **(A)** The experimental group shows the DDM filling the tooth extraction socket. **(B)** The control group shows a blood clot filling the tooth extraction socket.

All patients underwent tooth extractions performed by the same surgeon, who used minimally invasive tooth forceps and administered local anesthesia with 4% articaine. The dental professional meticulously removed soft tissues and foreign objects, including dental calculus and restorative materials. The crown enamel and cementum were subsequently removed using a turbine, while the remaining tooth tissues were crushed and filtered through a 1 mm sieve to obtain dentin particles. Dentin particles were then processed using the VacuaSonic^®^ System equipment (CosmoBioMedicare, Korea) to obtain DDM (**Figure 1**). In the experimental group, the extraction socket was solely filled with DDM, elevating it by 1 mm above the alveolar crest, while the control side was allowed to heal naturally (**Figure 2**).

Following the operation, it is advisable to apply a cold compress to both sides of the surgical area for 24 to 48 hours. Additionally, it is recommended to consume warm or cool foods two hours after the operation and adhere to the required precautions for post-tooth extraction care. Starting from the second day post-operation, it is suggested to rinse the mouth with diluted tinidazole (10-15 ml Tid) for f three days.

During the postoperative follow-up, measurements of the parameters were conducted by two experienced doctors, one being an oral and maxillofacial surgeon, and the other being a periodontist.

### Outcome evaluation

#### Postoperative pain and gingival condition

Pain intensity of patients on the 1st day postoperatively was assessed using the Visual Analogue Scale (VAS) method (**Table 1**) (23). Gingival congestion and swelling scores at the surgical site were evaluated and documented using the gingival condition classification during follow-up visits at 3 and 7 days postoperatively (**Table 2**) (24).

**Table 1:** VAS scoring criteria.

Pain Rating	Score	Pain Level
**painless**	0	Painless
**mild pain**	1-3	There is mild pain that the patient can tolerate
**moderate pain**	4-6	The patient suffers from pain and sleep disturbance, but it can be tolerated
**severe pain**	7-10	The patient has increasingly intense pain or the pain is unbearable, affects appetite, and affects sleep

**Table 2:** Gingival condition classification.

Grading	Colors	Swollen
**0**	Consistent with surrounding gingival color	Swelling is not obvious, consistent with the surrounding gingival, more stable
**1**	Heavier than surrounding gingival, but overly natural	Mild swelling, softer buccal flap
**2**	The gingival are dark red and brightly colored	Swelling is obvious

### The height of the buccal gingival margin after operation

**Figure 3:**
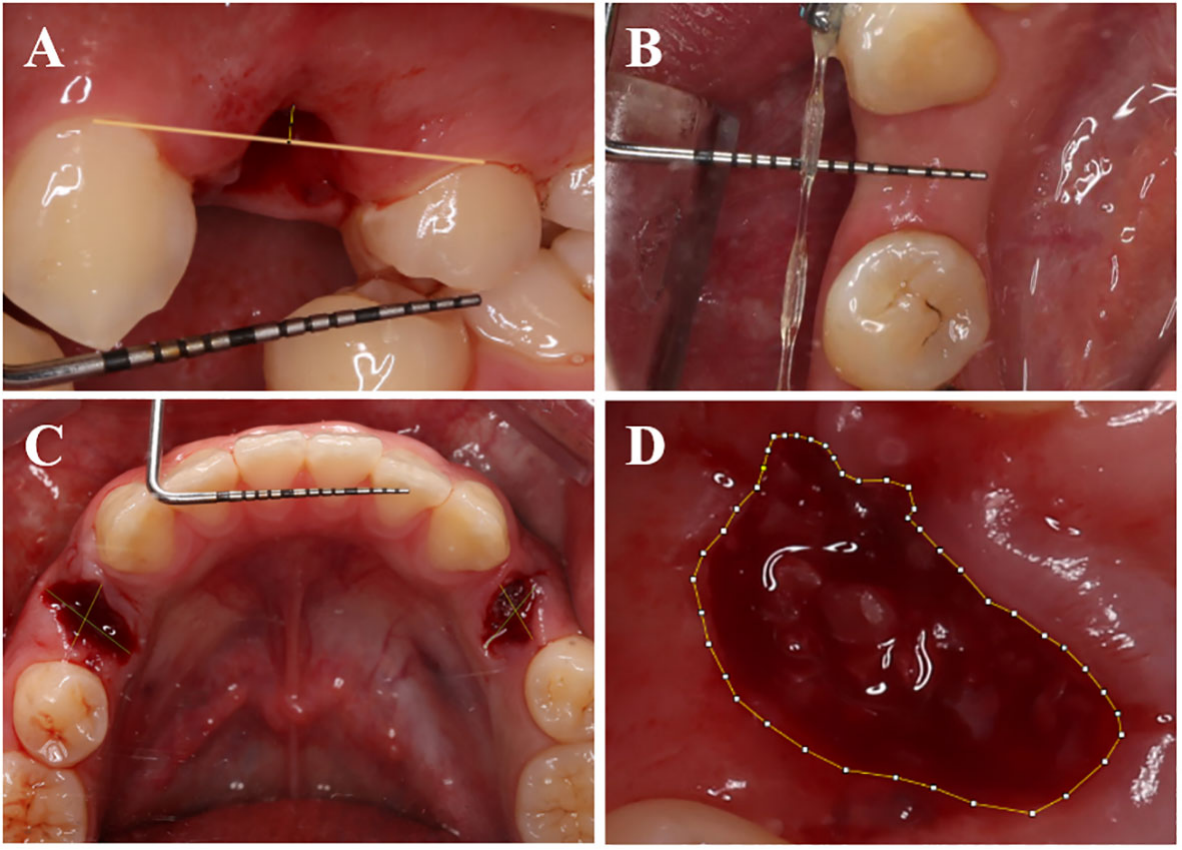
Measurement methods for certain parameters. **(A)** Capture photographs vertically aligned with the buccal side of the tooth extraction socket, and in Image J software, measure the postoperative buccal gingival margin height using the periodontal probe scale as a reference. **(B)** Utilize a periodontal probe to measure the buccal-lingual (palatal) width of the soft tissue on the alveolar crest postoperatively. **(C)** Capture photographs vertically aligned with the occlusal surface of the extraction socket, and in ImageJ software, use the periodontal probe scale as a reference to measure the postoperative extraction socket contour. **(D)** Use Image J software to trace the outline of the tooth extraction socket and calculate the healing area of the socket.

Immediately after the operation, photographs were taken of the buccal plane of the vertical extraction socket. Additional photographs were captured during the follow-up visits on the 3rd and 30th days (**Figure 3A**). The height of the buccal gingival margin was measured using Image J software (National Institutes of Health, American).

### The buccal-lingual (palate) width of the soft tissue of the alveolar ridge after operation

During the 30-day follow-up visit after operation, a periodontal probe was employed to measure and document the buccal-lingual (palate) dimension of the alveolar ridge’s soft tissue (**Figure 3B**).

### The dimensions of the socket contour

Images were captured of the occlusal surface of the extraction socket right after the operation, as well as on the 7th, 14th, and 30th days. The approach employed to assess the dimensions of the mesial-distal (M-D) and buccal-lingual (B-L) extraction sockets post-operation was adopted from Suttapreyasri et al. (25) (**Figure 3C**).

### The healing area and healing rate of tooth extraction socket after the operation

**Figure 4:**
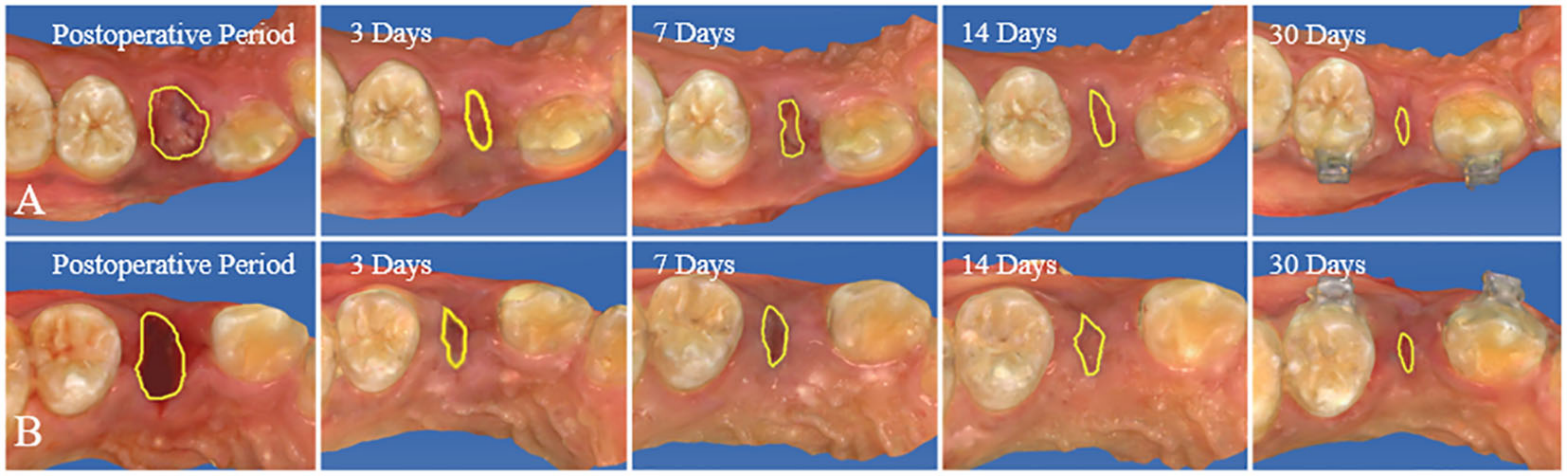
Utilize Image J software to digitally outline the extraction socket in intraoral optical impression images and assess the postoperative healing rate of the extraction socket. **(A)** Control group. **(B)** Experiment group.


Researchers employed a CEREC chairside system scanner (Sirona, Germany) to capture intraoral digital optical impressions right after the operation, as well as at postoperative days, 7, 14, and 30. The extraction socket’s area was quantified utilizing Image J software, with scale adjustments applied (**Figure 3D**). Subsequently, the healing rate of the extraction socket was computed employing a specific formula (1) (26) (**Figure 4**).

(1) $$\text{Healing rate}=\frac{\text{immediate extraction socket area}-\text{extraction socket area}}{\text{immediate extraction socket area}}\times 100\%$$

### Histopathological examination

Using forceps, fibrous tissue samples measuring approximately 1mm × 1mm were taken from the surface of the extraction sockets of some patients in the experimental group 3 days after the operation. In contrast, blood clots from the extraction sockets were obtained for the control group and subsequently placed separately in 10% formaldehyde fixative solution for fixation, with a fixation time of 24 hours. Following specimen fixation, a stepwise process was employed including rinsing in running water, automated dehydration using a tissue specimen dehydrator, xylene-based transparency, wax immersion, paraffin embedding, and tissue sectioning using a rotary microtome (with an average thickness of 3μm). Subsequently, Alcian Blue staining was conducted followed by microscopic observation.

### Statistical analysis

All collected data underwent statistical analysis using SPSS 26.0 software (SPSS, USA). Normality and variance homogeneity of the data were assessed using the Shapiro-Wilk and Levene tests. In cases of ordinal data, the nonparametric rank-sum test for two independent samples was applied. If the assumptions of a normal distribution and homogeneity of variances are met, a paired t-test will be used. In cases where the data does not follow a normal distribution, the nonparametric Wilcoxon signed-rank test will be employed. A *P*-value below 0.05 denoted statistical significance in the observed discrepancies.

## Results

**Figure 5:**
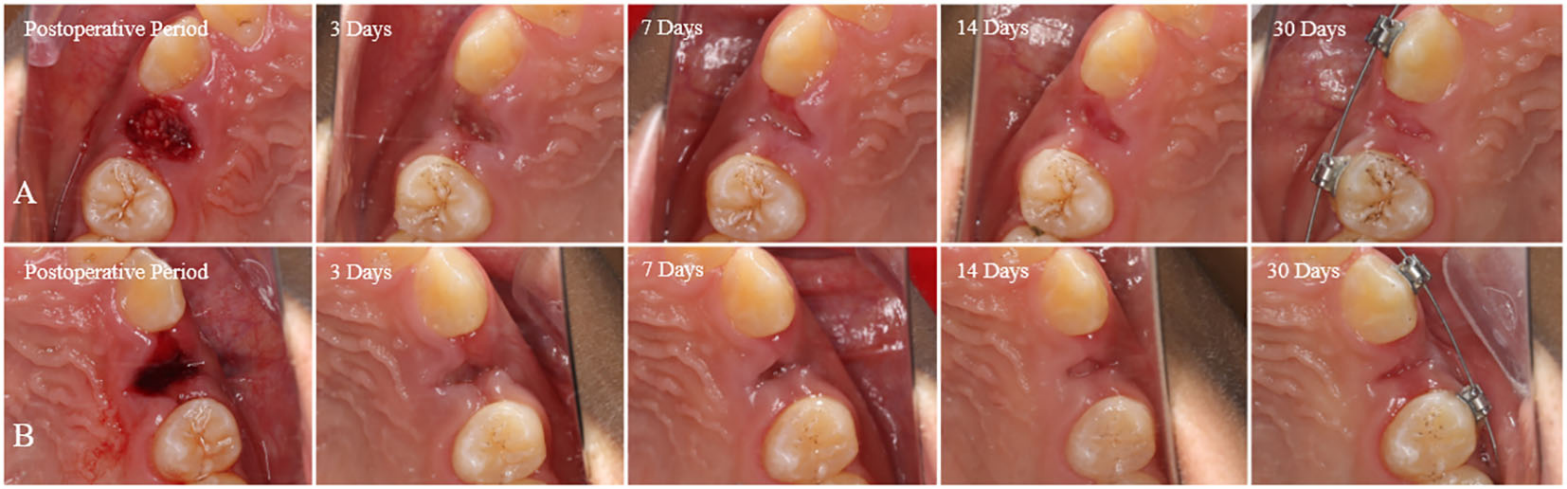
From immediately postoperative to 30 days postoperative, both the experimental and control groups show good healing of the extraction sockets, without any complications or evident bone graft material loss. **(A)** Control group. **(B)** Experiment group.

**Figure 6:**
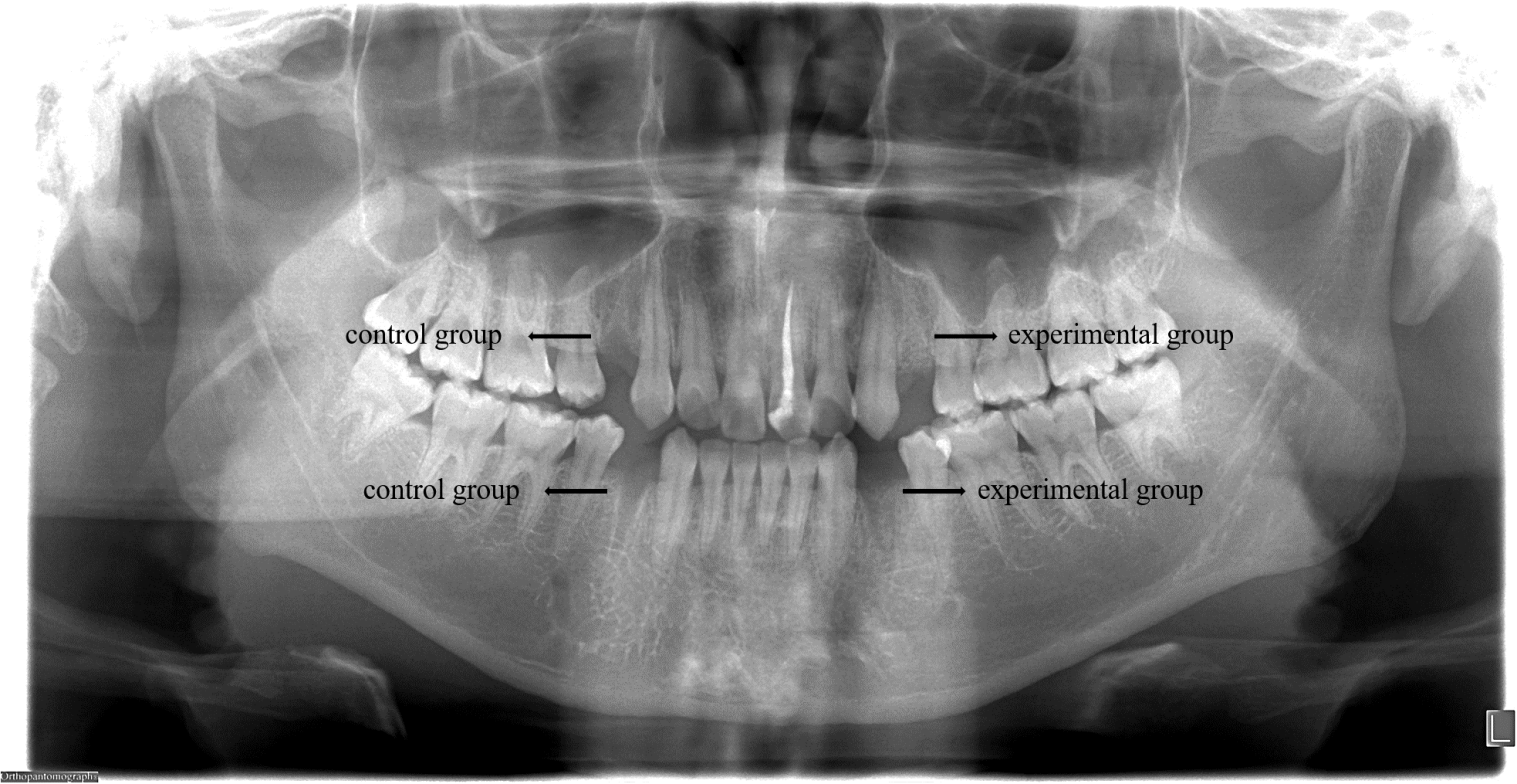
At 30 days postoperative, radiographic images reveal the presence of DDM in the extraction sockets of the experimental group, preserving the outline of hard tissues, while the control group shows signs of bone resorption.

This study included 14 patients requiring bilateral removal of maxillary or mandibular premolars for orthodontic treatment, involving a total of 44 premolars. Among participants, there were 4 males and 10 females, with an average age of 22.1 ± 8.1 years. All patients received regular follow-up, and postoperative healing of the extraction sockets was excellent, with no complications or significant bone graft material loss observed (**Figure 5**). In the radiographic images at 30 days postoperative, DDM within the extraction sockets of the experimental group can be observed, preserving the outline of hard tissues (**Figure 6**).

### Postoperative VAS pain assessment

One day post-operation, the experimental group reported 9 painless sites, 11 sites with mild pain, 2 sites with moderate pain, and no sites with severe pain. In contrast, the control group displayed 5 painless sites, 12 sites with mild pain, 4 sites with moderate pain, and 1 site with severe pain. Despite these differences in pain levels, statistical analysis utilizing **Table 3** revealed no significant variation in pain levels between the experimental and control groups (*P*>0.05).

**Table 3:** Postoperative VAS Pain Assessment.

Pain Grade	Sites		Total	Rank	Average Rank	Rank-sum	
	Experiment	Control				Experiment	Control
**0**	9	5	14	1~14	7.5	67.5	37.5
**1**	11	12	23	15~37	26	286	312
**2**	2	4	6	38~43	40.5	81	162
**3**	0	1	1	44~44	44	0	44
	22	22	44			434.5	555.5

### Gingival congestion and swelling

**Figure 7:**
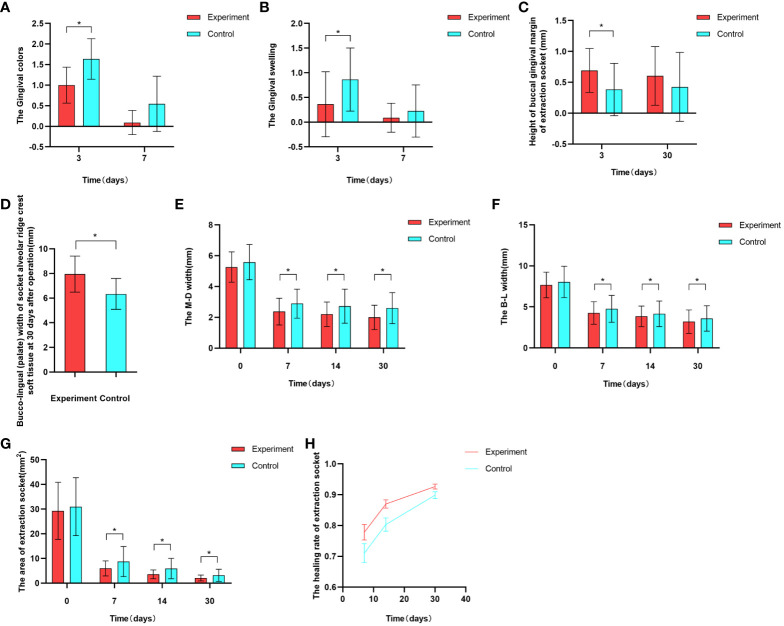
Graphical representation of some experimental results. **(A)** Comparison of gingival color between the experimental and control groups at 3 and 7 days postoperative . **(B)** Comparison of gingival swelling between the experimental and control groups at 3 and 7 days postoperative. **(C)** Comparison of buccal gingival margin height in the experimental and control groups at 3 and 30 days postoperative. **(D)** Comparison of buccal-lingual (palate) width of soft tissue on the alveolar crest in the experimental and control groups at 30 days postoperative. **(E, F)** Comparison of extraction socket contours between the experimental and control groups at immediately postoperative, 7 days, 14 days, and 30 days postoperative. **(G, H)** Comparison of extraction socket healing area and healing rate between the experimental and control groups at immediately postoperative, 7 days, 14 days, and 30 days postoperative.

After tooth extraction, the gingival color was evaluated at 3 days post-operation in both the experimental and control groups. Within the experimental group, 2 sites exhibited consistent gingival color with the surrounding area, while 18 sites displayed redness and 2 sites appeared slightly dull in color. In contrast, within the control group, 8 sites were red and 14 sites were slightly dull in color. The disparity between the two groups was found to be statistically significant (*P*<0.05). After a week of extraction, among the experimental group, 18 sites demonstrated gingival color matching the surrounding area, and 4 sites exhibited a reddish hue. Meanwhile, in the control group, 14 sites showcased harmonized color with the surrounding gingiva, and 8 sites appeared reddish. Nevertheless, the variation between the two groups lacked statistical significance (*P*>0.05) (**Figure 7A**).

At 3 days postoperative, both experimental and control groups exhibited gingival swelling. In the experimental group, 16 sites displayed no notable swelling, while 4 sites showed slight swelling and 2 sites exhibited evident swelling. Conversely, the control group had 6 sites without notable swelling, 13 sites with slight swelling, and 3 sites with evident swelling. The disparity between the groups was statistically significant (*P*<0.05). After a week, within the experimental group, 20 sites showed no apparent swelling and slight swelling was observed in 2 sites. In contrast, the control group had 18 sites without apparent swelling, 3 sites with slight swelling, and 1 site with evident swelling. However, the distinction between the two groups lacked statistical significance (*P*>0.05) (**Figure 7B**).

### The gingival height of buccal side of the socket

Three days postoperative, the gingival margin height on the buccal side of the extraction socket was 0.696 ± 0.345 in the experimental group, and 0.384 ± 0.425 in the control group, with a statistically significant difference between the two groups (*P*<0.05)(**Figure 7C**).

At 30 days postoperative, measurements of the buccal gingival margin were taken in both the experimental group (0.604 ± 0.475) and the control group (0.427 ± 0.558). While the experimental group displayed a slightly higher measurement, the disparity between the two groups lacked statistical significance (*P*>0.05)(**Figure 7C**).

### The buccal-lingual(palate)width of soft tissue in an alveolar crest of the extraction socket

At 30 days postoperative, the buccal-lingual (palatal) width of the alveolar crest soft tissue in the experimental group was 7.949 ± 1.460, while in the control group, it was 6.341 ± 1.257. The difference between the two groups was statistically significant(**Figure 7D**).

### The dimensions of the socket contour

Initially, there was no notable difference between the experimental and control groups following the operation (*P*>0.05). However, after 7, 14, and 30 days, the M-D and B-L widths of the experimental group were significantly lower than those of the control group.The difference was statistically significant (*P*<0.05) (**Table 4**, **Figures 7E, F**).

**Table 4:** The dimensions of the socket contour (mm, mean ± standard).

Times	M-D(mm)			B-L(mm)			
	experimental group	Control group	*P-*Value		experimental group	Control group	*P*-Value
Immediately after operation	5.265 ± 0.984	5.585 ± 1.142	>0.05		7.673 ± 1.559	8.045 ± 1.918	>0.05
7 days after operation	2.370 ± 0.869	2.888 ± 0.943	<0.05		4.258 ± 1.367	4.764 ± 1.637	<0.05
14 days after operation	2.195 ± 0.793	2.726 ± 1.106	<0.05		3.852 ± 1.255	4.161 ± 1.557	<0.05
30 days after operation	1.994 ± 0.792	2.595 ± 1.008	<0.05		3.207 ± 1.427	3.599 ± 1.546	<0.05

### The healing area of tooth extraction socket

Immediately after operation, the healing area of the extraction socket in the experimental group was 29.297 ± 11.586, while in the control group, it was 30.977 ± 11.746. The difference between the two groups had no statistical significance (*P*>0.05). However, on postoperative days 7, 14, and 30, the extraction socket areas of the experimental group were 5.352 (3.720, 7.792), 3.451 (2.308, 4.095), and 1.826 (1.005, 2.930), respectively, while those of the control group were 5.650 (4.673, 12.691), 3.873 (2.997, 7.977), and 2.472 (1.777, 4.021). The differences between the two groups were statistically significant in all cases (*P*>0.05)(**Figure 7G**).

### The healing rate of tooth extraction socket

At postoperative days 7, 14, and 30, the healing rates of the extraction sockets in the experimental group were 77.82 ± 11.81, 86.98 ± 6.29, and 92.66 ± 3.73, respectively, while in the control group, they were 71.05 ± 14.47, 80.33 ± 10.12, and 89.86 ± 5.13. The differences between the two groups were statistically significant in all cases (*P*<0.05) (**Figure 7H**).

### Histological assessment

**Figure 8:**
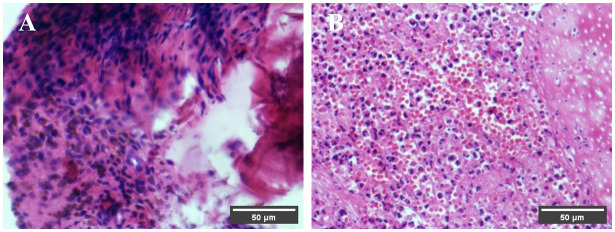
Pathological findings at 3 days postoperative. Under the microscope, the DDM particles in the experimental group exhibit a loose porous structure, and neutrophil infiltration is observed around both the DDM particles in the experimental group and the blood clots in the control group. **(A)** Control group. **(B)** Experiment group.

At 3 days postoperative, the results of Alcian Blue-hematoxylin and eosin staining, as shown in **Figure 8**, revealed that in the experimental group under the microscope, loose porous structures of DDM particles could be observed, with significant infiltration of neutrophils in the vicinity, and neutrophil infiltration was also observed inside dentinal tubules; while in the control group, only a small amount of neutrophil infiltration was observed.

## Discussion

After a tooth extraction, the alveolar bone experiences a lack of typical physiological stimulation. Consequently, the residual alveolar ridge undergoes irreversible resorption, potentially resulting in bone loss and recession of soft tissue at the extraction site (1). This phenomenon is notably pronounced within orthodontic treatment, where tooth extractions commonly become necessary for addressing concerns such as crowded dentition, protrusion, and severe caries (27). Such effects might potentially influence the pace of tooth movement in orthodontic treatment and the extent of root resorption observed in adjacent teeth, along with the emergence of gingival clefts in subsequent orthodontic interventions (5, 28–30).

Previous research has employed diverse bone substitute materials such as deproteinized bovine bone mineral, nanocrystalline hydroxyapatite, allograft bone, bone ceramic, and BMP2-functionalized biomimetic calcium phosphate grafts. These materials aim to mitigate the effects of tooth extraction on subsequent orthodontic procedures (30–35). However, the predominant focus of research in this domain lies in areas such as tooth movement, space closure, root resorption, and gingival clefts. Regrettably, only a limited array of studies had delved into the ramifications of these procedures on the healing of soft tissue.

In 1967, Urist postulated that rabbit dentin possesses the capability to initiate bone formation by orchestrating the conversion of connective tissue into bone *via* the process of endochondral osteogenesis (36, 37). Since then, a multitude of animal and clinical studies have been undertaken to validate the biocompatibility, biodegradability, osteoinductive, and osteoconductive properties of DDM (18, 38–41).

The present study utilized DDM for alveolar ridge preservation after orthodontic extraction. Initially, the focus centered on observing its promotional effect on gingival soft tissue. Employing a split-mouth design, premolars from a single patient’s dental arch were randomly assigned to either an experimental or control group. This design proves advantageous, facilitating equitable inter-group comparisons and mitigating the influence of external variables, such as diet and oral hygiene, on experimental outcomes. The experiment divulged no statistically significant variation in socket width and the area between the experimental and control groups post tooth extraction, signifying parity in initial conditions and the elimination of selection bias. Consequently, subsequent comparisons can be executed with a high degree of confidence.

This study excluded methods like employing a collagen membrane, connective tissue graft, and sutures to close the socket when applying DDM to ARP following orthodontic extractions. This decision was rooted in the research’s objective to directly observe the impact of DDM on gingival soft tissue, unobstructed by external variables. Consequently, the alveolar socket was left unsealed. Nevertheless, a panoramic radiograph captured 30 days post-operation depicted the persistent high-density filling image of DDM at the alveolar ridge level (**Figure 5B**). This observation implies that DDM can be effectively retained within ARP even in the absence of alveolar socket sealing. This finding is in line with the research conducted by Lim (22), Brkovic (42), and Saito (43), indicating that the open healing method of ARP, which involves no initial wound closure, can successfully preserve bone substitute materials and facilitate the healing of both soft and hard tissues.

Complications such as wound bleeding, surgical site swelling, and pain are common after tooth extraction (44). The study utilized a minimally invasive approach for extracting premolar teeth. On the third day post-operation, the experimental group exhibited lighter gingival color and reduced swelling in contrast to the control group. These findings suggest that DDM can effectively mitigate the inflammatory response during the initial stages of soft tissue healing. This effect is likely attributed to the presence of growth factors, such as TGF-β and VEGF, within DDM, which have the potential to suppress the local inflammatory response (40, 45, 46). The pathological sections confirmed that the inflammatory response process in the experiment extraction socket healing was similar to that of the control group 3 days after operation. This finding is consistent with Morikawa (47), Pikuła (48), and Zarei (49) research, which suggests that growth factors can accelerate soft tissue healing. On the 7th day post-operation, there was no notable contrast in gingival color and swelling between the experimental and control groups. This could be attributed to the gradual reduction of gingival soft tissue inflammation on the 7th-day post-operation, indicating that both groups had achieved secondary healing (50).

In this study, socket preservation was performed using DDM grafts, and the results were evaluated on the 3rd and 30th day. The findings indicate that the experimental group was able to maintain the buccal gingival margin height better than the control group, suggesting that DDM applied in ARP can reduce gingival recession. Other studies conducted by scholars have also demonstrated that DDM implantation in the socket can help maintain the height of the buccal bone plate, thereby preserving the width of the keratinized gingiva (51). After 30 days of socket preservation using DDM grafts, the experimental group showed a larger width of the soft tissue buccal-lingual (palate) of the alveolar ridge crest compared to the control group. This finding is consistent with previous studies by Del and Elfana (52, 53). DDM is an effective bone graft material for ARP as it helps maintain the width of the buccal bone plate of the extraction socket, which in turn preserves the fullness of the gingival and prevents the collapse of the buccal soft tissue.

The study findings reveal that at immediate, 7-day, 14-day, and 30-day post-operation intervals, the mesial-distal and buccal-lingual dimensions of the extraction socket were narrower in the experimental group compared to the control group. Furthermore, the healing rate of extraction sockets was notably greater in the experimental group, and the difference was statistically significant. This outcome can be ascribed to the presence of pivotal growth factors such as TGF-β, FGF, VEGF, EGF, and PDGF within DDM, which wield a pivotal role in the initial healing of soft tissues. The presence of these growth factors stimulates the recruitment of fibroblasts to the site of injury, accelerating their proliferation and expediting the deposition of the extracellular matrix (15, 49). Rinastiti (54) demonstrated the histological impact of transplanting the human amniotic membrane onto rabbit gingival wounds. This study revealed that the amniotic membrane effectively accelerates granulation tissue formation in gingival wounds through a rapid increase in fibroblast population and vascularization. The presence of bFGF, EGF, TGF-β, IL-1, and other growth factors within the amniotic membrane likely contributes to the expedited healing of gingival wounds. These growth factors possess the capacity to initiate fibroblast proliferation and promote neovascularization in gingival wounds. Stephan’s systematic review further substantiates the crucial role of growth factors in the wound-healing process (55). As bone graft materials play a role in the immune microenvironment during osteogenesis, the immune microenvironment is a complex environment composed of various immune cells, growth factors, extracellular matrix, and related signaling molecules, and these components play an important role in tissue regeneration and repair (56–58).

Several limitations of this study merit attention. Firstly, potential patient-related factors influencing wound healing were not accounted for. Subsequent research endeavors will delve into assessing the influence of DDM on gingival healing across diverse age groups. Secondly, the scope of histological analysis encompassed merely 4 of the 22 sites, potentially necessitating a broader sample and extended observation duration in future investigations. Thirdly, the specific demineralization rate and particle size of DDM were unreported. Therefore, a more comprehensive exploration of the physical and chemical attributes of DDM is imperative for optimizing its clinical efficacy. Furthermore, future investigations will compare the impact of various graft materials with DDM on gingival healing.

## Conclusions

This study demonstrates that the use of DDM in alveolar ridge preservation following orthodontic extraction can decrease the early inflammatory response of gingival tissue healing, accelerate the healing of gingival tissue, and maintain the contour of the tissue.

## Data availability statement

The raw data supporting the conclusions of this article will be made available by the authors, without undue reservation.

## Ethics statement

The studies involving humans were approved by institutional review board of School and Hospital of Stomatology, Fujian Medical University. The studies were conducted in accordance with the local legislation and institutional requirements. Written informed consent for participation in this study was provided by the participants’ legal guardians/next of kin.

## Author contributions

JG: Writing – original draft, Writing – review & editing. XX: Writing – original draft, Writing – review & editing. DP: Writing – original draft. BZ: Writing – original draft. KL: Writing – original draft. SW: Writing – original draft. WZ: Writing – review & editing. MZ: Writing – review & editing. JY: Writing – review & editing.
