# Corrigendum: Demineralized dentin matrix promotes gingival healing in alveolar ridge preservation of premolars extracted for orthodontic reason: a split-mouth study

**DOI:** 10.3389/fendo.2023.1357769

**Published:** 2024-01-16

**Authors:** Xiaofeng Xu, Dongsheng Peng, Bowei Zhou, Kaijin Lin, Siyi Wang, Wei Zhao, Minqian Zheng, Jin Yang, Jianbin Guo

**Affiliations:** ^1^ Fujian Key Laboratory of Oral Diseases & Fujian Provincial Engineering, Research Center of Oral Biomaterial & Stomatological Key Lab of Fujian College and University, School and Hospital of Stomatology, Fujian Medical University, Fuzhou, China; ^2^ Stomatological Key Laboratory of Fujian College and University, School and Hospital of Stomatology, Fujian Medical University, Fuzhou, China; ^3^ Department of Stomatology, Affiliated Hospital of Putian University, Putian, China; ^4^ Department of Stomatology, Fujian Maternity and Child Health Hospital, Affiliated Hospital of Fujian Medical University, Fuzhou, China; ^5^ Department of Stomatology, Fujian Obstetrics and Gynecology Hospital, Fuzhou, China; ^6^ Research Center of Dental and Craniofacial Implants, Fujian Medical University, Fuzhou, China

**Keywords:** demineralized dentin matrix, alveolar ridge preservation, gingival healing, bone graft materials, orthodontics

**Incorrect Affiliation**

In the published article, there was an error in affiliations 1 and 2. For the error in affiliation 1, instead of “Fujian Provincial Engineering Research Center of Oral Biomaterial, Fujian Medical University, Fuzhou, China”, it should be “Fujian Key Laboratory of Oral Diseases & Fujian Provincial Engineering, Research Center of Oral Biomaterial & Stomatological Key Lab of Fujian College and University, School and Hospital of Stomatology, Fujian Medical University, Fuzhou, China”.

For the error in affiliation 2, instead of “School and Hospital of Stomatology, Fujian Medical University, Fuzhou, China”, Stomatological Key Laboratory of Fujian College and University, School and Hospital of Stomatology, Fujian Medical University, Fuzhou, China”.

The authors apologize for these errors and state that they do not change the scientific conclusions of the article in any way. The original article has been updated.

