# Supplementary figures and images for: Demineralized dentin matrix promotes gingival healing in alveolar ridge preservation of premolars extracted for orthodontic reason: a split-mouth study
Source: Front Endocrinol (Lausanne). 2023 Oct 19;14:1281649. doi: 10.3389/fendo.2023.1281649 (PMC10622762; doi:10.3389/fendo.2023.1281649)

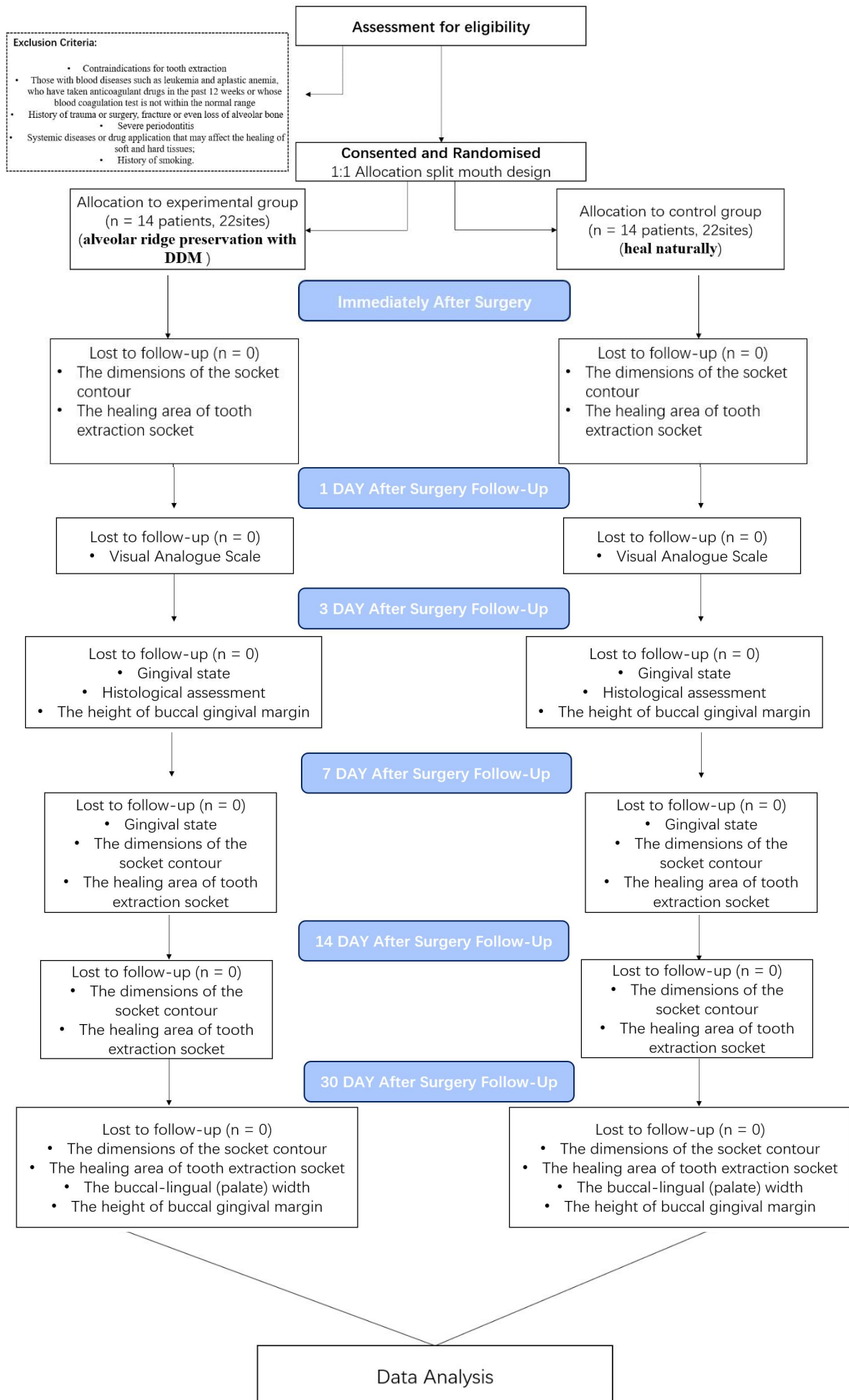

Supplement: Supplementary file 1 [file DataSheet_1.pdf]
